# The ongoing antibiotic resistance and carbapenemase encoding genotypes surveillance. The first quarter report of the INVIFAR network for 2024.

**DOI:** 10.1371/journal.pone.0319441

**Published:** 2025-04-16

**Authors:** Claudia Adriana Colín-Castro, Luis Esaú López-Jácome, María José Rodríguez-García, Melissa Garibaldi-Rojas, Fabián Rojas-Larios, María del Rosario Vázquez-Larios, Sandra Quintana-Ponce, Rafael Franco-Cendejas, Adolfo Gómez-Quiróz, Patricia Rodríguez-Zulueta, Thalia Rosado-Espinosa, Luis Javier Quintanilla-Cazares, Consuelo Velázquez-Acosta, Pablo Hernan Sandoval-Villaseñor, Juan Pablo Mena-Ramírez, Elena Victoria Choy-Chang, María Fernanda González-Lara, Bernardo A. Martínez-Guerra, Enrique Bolado-Martínez, Laura Karina Aviles-Benítez, José Manuel Feliciano-Guzmán, Carlos Antonio Couoh-May, Talia Pérez-Vicelis, Aldo Rafael Silva-Gamiño, Luis Raúl Rivera-Garay, Norberta Vianey Navarro-Vargas, Maricruz Gutiérrez-Brito, César Adame-Álvarez, Sandra María Suárez-Moreno, Bertha Alicia Pérez-Vega, Laura Isabel López-Moreno, Guillermo Jacobo-Baca, Mario Galindo-Méndez, María Bertha Ballesteros-Silva, Elda Gabriela Vázquez-Narvaez, Nicolás Rogelio Eric Barlandas-Rendón, Dora Elia Rodríguez-Balderas, Juan de Dios Castañeda-Duarte, Cecilia Padilla-Ibarra, Víctor Hugo Peralta-Peñúñuri, Alma Denia López-Vázquez, María Asunción Santiago-Calderón, Ángela Cecilia Valtierra, Paulina Fabiola González-Melgoza, Luis Gerardo Medina-Navarro, Christian Daniel Mireles-Dávalos, Ibis De-la-Cruz-Hernández, Guadalupe Soledad Huirache-Villalobos, Ernesto Mario Aguilar-Trejo, María del Rocío López-Álvarez, Elvira Garza-González

**Affiliations:** 1 Instituto Nacional de Rehabilitación Luis Guillermo Ibarra Ibarra, Mexico City, Mexico; 2 Universidad Autónoma de Guerrero, Chilpancingo, Guerrero, Mexico; 3 Departamento de Bioquímica y Medicina Molecular, Facultad de Medicina. Monterrey, Nuevo León. Mexico; 4 Universidad de Colima, Colima, Colima, Mexico; 5 Hospital Regional Universitario de los Servicios de Salud del Estado de Colima, Colima, Colima, Mexico; 6 Instituto Nacional de Cardiología Ignacio Chávez, Mexico City, Mexico; 7 Laboratorio de Microbiología. Hospital Civil de Guadalajara, Guadalajara, Jalisco. Mexico; 8 Hospital General Dr. Manuel Gea González, Mexico City, Mexico; 9 Hospital Ángeles Valle Oriente. Monterrey, Nuevo León, Mexico; 10 Instituto Nacional de Cancerología, Mexico City, Mexico; 11 Hospital General de Zona No. 21, IMSS, Tepatitlan de Morelos, Jalisco, Mexico; 12 Hospital General de Zona No.1, Nueva Frontera. IMSS, Tapachula, Chiapas, Mexico; 13 Departamento de Infectología. Instituto Nacional de Ciencias Médicas y Nutrición Salvador Zubirán, Mexico City, Mexico,; 14 Universidad de Sonora, Hermosillo, Sonora, Mexico; 15 Hospital Infantil de Morelia, Morelia, Michoacán, Mexico; 16 Hospital de Especialidades Pediátricas, Tuxtla Gutiérrez, Chiapas, Mexico; 17 Hospital General Dr. Agustín O´Horan, Mérida, Yucatán, Mexico; 18 Hospital de Alta Especialidad Bicentenario de la Independencia. ISSSTE, Tultitlán, Estado de México, Mexico; 19 Hospital Ángeles Morelia, Morelia, Michoacán, Mexico; 20 Hospital Regional de Alta Especialidad del Bajío, León, Guanajuato, Mexico; 21 Hospital General Presidente Lázaro Cárdenas del Río. ISSSTE, Chihuahua, Chihuahua. Mexico; 22 Hospital del Niño Poblano, San Andrés Cholula, Puebla, Mexico; 23 Centenario Hospital Miguel Hidalgo, Aguascalientes, Aguascalientes, Mexico; 24 Laboratorio DIPROMI, Morelia, Michoacán, Mexico; 25 Hospital Ángeles Chihuahua, Chihuahua, Chihuahua. Mexico; 26 Hospital Galenia. Cancún, Quintana Roo. Mexico; 27 Centro Universitario de Salud, Universidad Autónoma de Nuevo León, Monterrey, Nuevo León, Mexico; 28 Laboratorio Galindo, Oaxaca, Oaxaca, Mexico; 29 Centro de Diagnóstico Microbiológico, Morelia, Michoacán. Mexico; 30 Hospital General Dr. Miguel Silva, Morelia, Michoacán, Mexico; 31 Laboratorio BIOCLIN, Chilpancingo, Guerrero, Mexico; 32 Hospital del Niño Dr. Federico Gómez Santos, Saltillo, Coahuila, Mexico; 33 Centro Médico Dr. Ignacio Chávez, Hermosillo, Sonora, Mexico; 34 Hospital General del Estado de Sonora, Hermosillo, Sonora, Mexico; 35 Hospital General Dr. Fernando Ocaranza, Hermosillo, Sonora, Mexico; 36 Centro Integral de Atención a la Salud Sur. ISSSTE, Hermosillo, Sonora, Mexico; 37 Hospital General de Zona 1. IMSS, Oaxaca, Oaxaca, Mexico; 38 Hospital General de León, León, Guanajuato, Mexico; 39 Laboratorio de análisis clínicos y microbiológicos Futura Médica, Morelia, Michoacán, Mexico; 40 Hospital General de Silao, Silao, Guanajuato, Mexico; 41 Instituto Nacional de Enfermedades Respiratorias Ismael Cosio Villegas, Mexico City, Mexico; 42 Hospital General de Zona 46. IMSS, Villahermosa, Tabasco, Mexico; 43 Laboratorio Clínico y Bacteriológico La Barca, Jalisco, Mexico; 44 Laboratorio Biodiagnostics. Apizaco, Tlaxcala, Mexico; 45 Hospital Regional General Ignacio Zaragoza, Mexico City, Mexico,; 46 Centro Universitario de los Altos (CUALTOS), Universidad de Guadalajara, Tepatitlán de Morelos, Jalisco, Mexico; 47 Departamento de Biología, Facultad de Química. Universidad Nacional Autónoma de México, Mexico City, Mexico; University of Tripoli, Libya

## Abstract

**Introduction:**

Antimicrobial resistance surveillance plays an important role in generating information about the prevalence of resistant microorganisms. In this study, we summarize a surveillance of antimicrobial resistance and carbapenemase-encoding genes for selected pathogens in Mexican healthcare centers.

**Methods:**

Databases of identification and susceptibility results collected from January 1 to March 31, 2024, from forty-one centers were gathered and analyzed using the WHONET software. Some relevant gram-negatives and gram-positives, which were isolated from relevant clinical specimens were included. Isolates were stratified by patient´s age, clinical specimens, and site of attention, and were classified as multidrug-resistant (MDR). Clinical isolates were collected from January 1 to June 30 and were genotyped for carbapenemase-encoding genes by a polymerase chain reaction test.

**Results:**

In total, 8 708 strains were included. *Escherichia coli* had a higher resistance to carbapenems (*p* < 0.05) in the 0–17 years group and *Klebsiella pneumoniae* (*p* = 0.017)*, Pseudomonas aeruginosa,* and *Acinetobacter baumannii* (*p* < 0.05) in the 18–59 years group. *P. aeruginosa* had higher resistance to ceftazidime-avibactam, ceftolozane-tazobactam, cefepime, and imipenem (*p* < 0.05) in the 18–59 years group. *K. pneumoniae* had the highest resistance to carbapenems (*p* < 0.05) and cefepime (*p* < 0.001) in clinical isolates recovered from blood. For *P. aeruginosa,* the highest resistance to cefepime (*p =* 0.012) and ceftazidime (*p* < 0.018) was seen in isolates from urine. For *Staphylococcus aureus*, a higher resistance was observed for cefoxitin in lower respiratory tract specimens (*p* < 0.05). *E. coli* had the highest resistance to carbapenems (*p* < 0.01), and *P. aeruginosa* for ceftazidime (*p =* 0.005), cefepime (*p =* 0.003), piperacillin-tazobactam (*p =* <0.01), IPM (*p =* 0.006), and meropenem (*p =* <0.01) in clinical isolates recovered from patients in the intensive care unit (ICU). For *K. pneumoniae,* the highest resistance to ertapenem was observed in clinical isolates from the ICU area (p < 0.035). Finally, 67.9% of *A. baumannii* and 53.8% of *E. coli* strains were Multidrug-resistant. *Candida albicans* isolated from blood had susceptibility to caspofungin 100% and 90.2% for voriconazole. Regarding *E. coli* non-susceptible to meropenem, 16 (59.2%) were carriers of *bla*_*NDM,*_ and the *bla*_KPC_ gene was detected in 2 (40%) strains of *K. pneumoniae.*

In conclusion, carbapenem resistance was higher for *E. coli* in the 0–17 years group and for *K. pneumoniae*, *P. aeruginosa,* and *A. baumannii* in the 18–59 years group. *K. pneumoniae* has the highest resistance to carbapenems in blood isolates and the ICU area. *E. coli* and *P. aeruginosa* had the highest carbapenem resistance in the intensive care unit. A high multidrug resistance was observed for *A. baumannii* and *E. coli* strains. A high susceptibility to caspofungin and voriconazole was observed for *Candida albicans* collected from blood.

## Introduction

Surveillance of antimicrobial resistance is an important tactic to address this global public health problem and is fundamental for the identification of emerging patterns and changes in the prevalence of drug-resistant bacteria [1–3].

Recently, the World Health Organization (WHO) identified and reclassified antibiotic-resistant pathogens into critical, high, and medium-priority groups to guide research and development and public health interventions. Carbapenem-resistant *Acinetobacter baumannii* and *Enterobacterales* are classified into critical priority groups. Carbapenem-resistant *Pseudomonas aeruginosa*, vancomycin-resistant *Enterococcus faecium*, and methicillin-resistant *Staphylococcus aureus* (MRSA) are classified into the high-priority group [4,5].

Another relevant emerging opportunistic bacterial pathogen is *Stenotrophomonas maltophilia*. This bacterial species has intrinsic resistance to multiple broad-spectrum antibiotics and is recognized as one of the most common causative agents of respiratory co-infections and bacteremia in critically ill COVID-19 patients [6–9].

Moreover, in 2022, the WHO published its first fungal pathogen priority list, with *Candida albicans* listed in the critical priority category [10]. This yeast is the most common cause of candidemia worldwide, and although antifungal resistance is not common, rates in non-sterile site recovered isolates seem to be increasing. Thus, there is a need for systematic surveillance of antifungal resistance [11,12].

Surveillance studies of antimicrobial resistance are needed in various geographic areas to identify relevant pathogens [3,13]. This work aims to summarize a three-month surveillance of antimicrobial resistance to selected pathogens in 2024 in Mexico.

## Materials and methods

### Participating centers and data obtention

Members of the Network for the Research and Surveillance of Drug Resistance (Red Temática de Investigación y Vigilancia de la Farmacorresistencia, INVIFAR in Spanish) were invited to participate. Forty-one centers from seventeen states of Mexico did so voluntarily (Aguascalientes, Chiapas, Chihuahua, Coahuila, Colima, Guanajuato, Jalisco, Mexico City, Mexico State, Michoacán, Nuevo León, Oaxaca, Puebla, Quintana Roo, Sonora, Tabasco and Yucatán). Personnel from these centers shared identification and susceptibility results of the clinical isolates recovered from January 1st to March 31st, 2024 (Each center’s information is outlined in Table 1).

**Table 1:** Characteristics of participating centers with hospitalization.

Center	State	Pu/Pr	No. beds	ICU beds	Type of Hospital
Laboratorio de Microbiología. Hospital Civil de Guadalajara	Jalisco	Pu	747	100	Spe
Instituto Nacional de Rehabilitación Luis Guillermo Ibarra Ibarra	Mexico City	Pu	222	15	Spe
Centenario Hospital Miguel Hidalgo.	Aguascalientes	Pu	218	32	Spe
Instituto Nacional de Ciencias Médicas y Nutrición Salvador Zubirán	Mexico City	Pu	212	14	Spe
Instituto Nacional de Cardiología Ignacio Chávez	Mexico City	Pu	208	28	Spe
Hospital de Alta Especialidad Bicentenario de la Independencia. ISSSTE	Mexico State	Pu	196	24	Spe
Hospital Regional de Alta Especialidad del Bajío	Guanajuato	Pu	184	25	Spe
Instituto Nacional de Enfermedades Respiratorias Ismael Cosio Villegas	Mexico City	Pu	161	15	Spe
Instituto Nacional de Cancerología	Mexico City	Pu	148	8	Spe
Centro Médico Dr. Ignacio Chávez. ISSSTE	Sonora	Pu	125	4	Spe
Hospital Ángeles Chihuahua	Chihuahua	Pr	125	6	Spe
Hospital Ángeles Valle Oriente	Nuevo León	Pr	102	8	Spe
Hospital Ángeles Morelia	Michoacán	Pr	55	12	Spe
Hospital Regional General Ignacio Zaragoza. ISSSTE	Mexico City	Pu	325	12	Gen
Hospital General Dr. Agustín O´Horan	Yucatán	Pu	296	30	Gen
Hospital General de León.	Guanajuato	Pu	260	29	Gen
Hospital General Dr. Miguel Silva	Michoacán	Pu	250	16	Gen
Hospital General de Zona 1. IMSS	Oaxaca	Pu	250	8	Gen
Hospital General de Zona 46. IMSS	Tabasco	Pu	190	18	Gen
Hospital General de Zona No.1, Nueva Frontera, IMSS	Chiapas	Pu	180	61	Gen
Hospital General del Estado de Sonora	Sonora	Pu	168	23	Gen
Hospital General Dr. Manuel Gea González	Mexico City	Pu	130	10	Gen
Hospital General Presidente Lázaro Cárdenas del Río. ISSSTE	Chihuahua	Pu	120	6	Gen
Hospital Regional Universitario de los Servicios de Salud del Estado de Colima	Colima	Pu	108	9	Gen
Hospital General Dr. Fernando Ocaranza. ISSSTE	Sonora	Pu	79	3	Gen
Hospital General de Zona No. 21 IMSS	Jalisco	Pu	73	13	Gen
Hospital General de Silao	Guanajuato	Pu	60	6	Gen
Hospital Galenia	Quintana Roo	Pr	46	14	Gen
Hospital Infantil de Morelia	Michoacán	Pu	130	10	Ped
Hospital del Niño Poblano	Puebla	Pu	102	15	Ped
Hospital de Especialidades Pediátricas	Chiapas	Pu	75	13	Ped
Hospital del Niño Dr. Federico Gómez Santos	Coahuila	Pu	44	13	Ped

Gen: general, ICU: intensive care units, Ped: pediatrics, Pr: private, Pu: public, Spe: specialties.

### Analysis of data

All databases were converted into the WHONET format using the Backlink tool. Converted files were analyzed using WHONET 2024 v24.7.3 [14].

During the analysis, data from patients was encrypted to protect personal information. Antimicrobial susceptibility tests (AST) results were collected from *Escherichia coli, Klebsiella pneumoniae, A. baumannii, P. aeruginosa, S. maltophilia, S. aureus*, and *E. faecium* isolated from blood, biopsies, abscesses, pleural fluid, urine, and lower respiratory tract (LRT) samples (bronchoalveolar lavage and endotracheal aspirate) were studied. *E. faecium* was not analyzed from LRT samples. AST results for blood-recovered *C. albicans* were included. Only those strains with genus-species identification certainties ≥95% were accepted. Duplicated samples were eliminated.

Antibiotics evaluated were amikacin (AK), amoxicillin-clavulanic acid (AMC), ampicillin (AMP), aztreonam (ATM), ampicillin-sulbactam (SAM), cefepime (FEP), cefoxitin (FOX), ceftazidime (CAZ), ceftazidime-avibactam (CZA), ceftolozane-tazobactam (CT), cefotaxime (CTX), ceftriaxone (CRO), cefuroxime (CXM), ciprofloxacin (CIP), clindamycin (CC), erythromycin (E), ertapenem (ETP), gentamicin (GN), gentamicin high levels (GNH), imipenem (IPM), linezolid (LZD), levofloxacin (LVX), meropenem (MEM), nitrofurantoin (NIT), norfloxacin (NOR), oxacillin (OXA), streptomycin high levels (STRH), tetracycline (TE), penicillin (PEN), piperacillin-tazobactam (TZP), trimethoprim-sulfamethoxazole (SXT), and vancomycin (VN). Categorical definitions (susceptible, intermediate, and resistant) were included for each antibiotic according to CLSI M100 34^th^ ed. breakpoint [15]. For *C. albicans*, caspofungin and voriconazole categorical definitions were used according to CLSI M27M24S Ed.3:2022 [16].

Drug susceptibility results were stratified according to the following age groups: 0–17 years, 18–59 years, and > 60 years. The drug susceptibility was also analyzed by type of specimen and patient location type, including emergency room (EME), intensive care unit (ICU), hospitalized non-ICU (INX), and outpatients (OUT).

Isolates were classified as multidrug-resistant (MDR), extensively drug-resistant (XDR), pan-drug-resistant (PDR), possible XDR, and possible PDR according to previously reported criteria [17].

### Carbapenemases encoding genes

As a result of the active surveillance by the INVIFAR network, the participating centers sent selected strains to the coordinating center for genotyping. For this study, strains collected in an extended 6-month period (from January 1, 2024, to June 30, 2024) were analyzed; first, carbapenem resistance was determined by the disk diffusion method, and intermediate or resistant strains were further analyzed. Strains were received and DNA was obtained by thermal lysis. End-point Polymerase chain reaction tests were performed for the most frequent carbapenemase-encoding genes: *bla*_*NDM-1*_*, bla*_*KPC*_*, bla*_*VIM*_*, bla*_*IMP*_*,* and *bla*_*OXA-48-like*_ in *E. coli* and *K. pneumoniae*; *bla*_*OXA-23*_ and *bla*_*OXA-24*_ in *A. baumannii;* and *bla*_*GES*_*, bla*_*VIM*_ and *bla*_*IMP*_*,* in *P. aeruginosa* strains [18].

### Analysis

Descriptive statistics were used to report frequencies. Chi-square or Fisher’s exact tests were used as appropriate to compare the frequencies of antibiotic resistance according to stratified groups (age group, clinical specimens, and sites of care). A two tailed *p* value ≤ 0.05 was considered significant. Statistical analysis was performed using the IBM SPSS Statistics 26 software [19].

### Ethics statement

This project was conducted under the approval assignation number 55/22 AC issued by the Research Committee from Instituto Nacional de Rehabilitación Luis Guillermo Ibarra Ibarra. The need for informed consent was waived by the ethics committee. The experiments were conducted according to the Declaration of Helsinki. Patient data was not used.

## Results

### Participating centers and resistance according to age

Among the participating centers, 31/41 (75.6%) belong to the Mexican public healthcare system, while the remaining 10/41 (24.4%) are affiliated to private care centers. The total number of data samples included in the study was 8 708 strains (4618 *E. coli*, 883 *K. pneumoniae*, 182 *A. baumannii*, 765 *P. aeruginosa*, 105 *S. maltophilia*, 559 *S. aureus*, 331 *E. faecium*, and 42 *C. albicans*).

**Fig 1:**
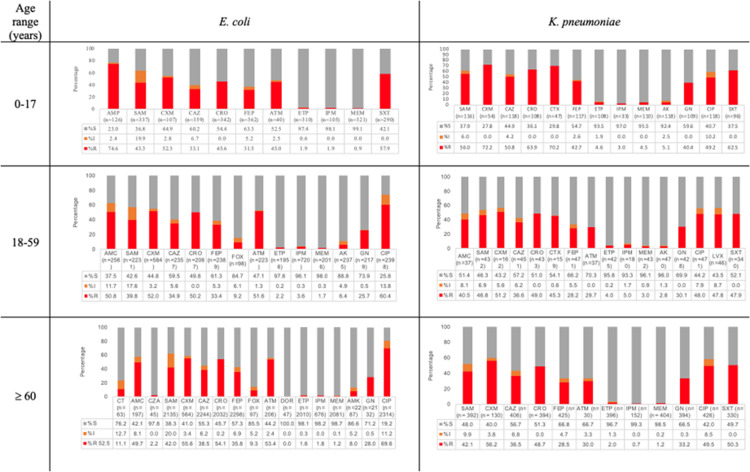
Distribution of antibiotic resistance according to age range of selected *Enterobacterales: E. coli* (n =**4 618) and** ***K. pneumoniae*** **(n**=**883).** Amikacin (AMK), amoxicillin-clavulanic acid (AMC), ampicillin (AMP), ampicillin-sulbactam (SAM), aztreonam (ATM), cefepime (FEP), cefotaxime (CTX), cefoxitin (FOX), ceftazidime (CAZ), ceftazidime-avibactam (CZA), ceftolozane-tazobactam (CT), ceftriaxone (CRO), cefuroxime (CXM), ciprofloxacin (CIP), doripenem (DOR), ertapenem (ETP), gentamicin (GN), imipenem (IPM), levofloxacin (LVX), meropenem (MEM), trimethoprim-sulfamethoxazole (SXT), S: susceptible; I: intermediate; R: resistant.

*A. baumannii* had the highest frequency of resistance to carbapenem (MEM 40.6%), and *E. coli* had the lowest resistance to these antibiotic class (0.9–1.9%) (Fig 1). In this group, antipseudomonal cephalosporin resistance in *P. aeruginosa* was 10% and 16% for FEP and CAZ, respectively. Additionally, for CT and CZA, resistance was near 2.4% for both combinations. The frequency of *Enterobacterales* including *K. pneumoniae* and *E. coli* with extended-spectrum ß-lactamases (ESBLs) was 60.6% and 48.6%, respectively.

**Fig 2:**
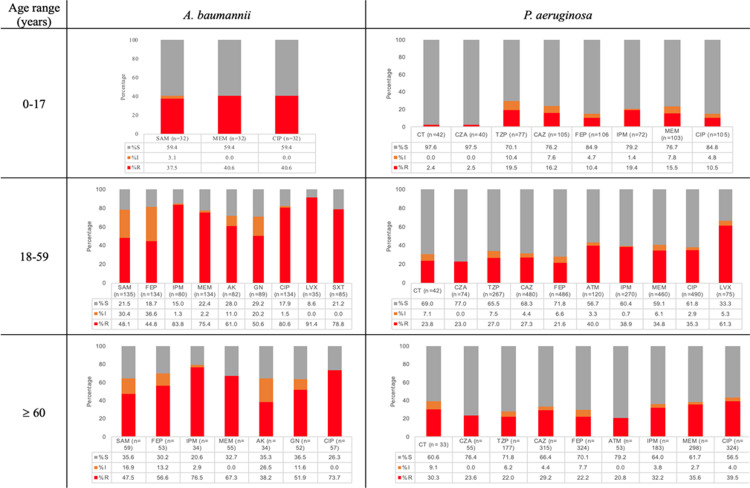
Distribution of antibiotic resistance according to the age range of selected non-fermenters: *A. baumannii* (n =**182). and** ***P. aeruginosa*** **(n**=**765).** Amikacin (AK), aztreonam (ATM), ampicillin-sulbactam (SAM), cefepime (FEP), ceftazidime (CAZ), ceftazidime-avibactam (CZA), ceftolozane-tazobactam (CT), ciprofloxacin (CIP), gentamicin (GN), imipenem (IPM), levofloxacin (LVX), meropenem (MEM), piperacillin-tazobactam (TZP), trimethoprim-sulfamethoxazole (SXT). S: susceptible; I: intermediate; R: resistant.

In the 18–59 years group*,* IPM and MEM resistance in *A. baumannii* was observed in 83.7% and 75.3%, respectively (Fig 2), and *P. aeruginosa* had resistance to CZA at 22.9% and CT at 23.8%. Moreover, for *E. coli*, within carbapenems, IPM had the higher resistance values (3.6%) compared to MEM (1.7%). *S. maltophilia* presented susceptibility to SXT of 75.3%.

In the > 60 years group, the highest resistance was observed in *A. baumannii* for IPM (76.4%), followed by ciprofloxacin (73.6%). Methicillin resistance *S. aureus* (MRSA) was documented in 32.10%; see Fig 1, Fig 2, and S1 Figure in S1 file.

When comparing age groups, *E. coli* had a higher frequency of ESBL-producing strains in the > 60 years group (*p* = 0.03) and a higher frequency of resistance to IPM (*p* < 0.05) in the group 0–17 years. For *K. pneumoniae,* a higher resistance was detected for CAZ, CRO, (*p* < 0.05), and SXT (*p =* 0.041) in the 0–17 years group. Furthermore, a higher resistance frequency was observed for IPM (*p* = 0.017) in the 18–59 years group. For *A. baumannii* and *P. aeruginosa,* resistance to MEM was more common in the 18–59 years group than in the other groups (*p* < 0.05). For CIP, *A. baumannii* had the highest resistance in the 18–59 group (*p* < 0.05), and *P. aeruginosa* (*p* < 0.05), *E. faecium* and *S. aureus* in the > 60 years group (*p* = 0.0001). A higher resistance was detected for CZA, CT, and FEP, as well as for IPM (*p* < 0.05) in the 18–59 years group. No differences were detected for VN resistance in *E. faecium* among the three groups. Resistance to VN in *S. aureus* was not found in any isolate (S1 Table).

### Resistance by clinical specimen

Among clinical isolates recovered from blood, more than half of *E. coli* and *K. pneumoniae* were ESBL producers (71.5% and 52.3%, respectively). *K. pneumoniae* had a 5.6% resistance to IPM and an 8.4% resistance to ETP and MEM. By contrast, *E. coli* had a resistance of 2.4% for MEM, 3.5% for ETP, and 3.6% for IPM. In *P. aeruginosa,* 5% were resistant to CZA, 3.2% were resistant to CT, while for MEM and IPM were resistant 25.2% and 28.2% respectively. For *A. baumannii*, resistance to MEM was 67.2%, and for IPM it was 73.3%. Fifteen percent of all *S. aureus* recovered from blood were defined as MRSA and resistance to VN was 0%. In this first approach of resistance to *Candida spp.* We received, from participant centers, only *C. albicans* recovered from bloodstream cultures. Antifungals included were VOR and CAS, no one strain (N=42) showed resistance to equinocandin CAS meanwhile resistance to VOR was 2.4%.

In LRT, 82.1% of *E. coli* and 60.4% of *K. pneumoniae* were ESBL producers. For *K. pneumoniae*, resistance to carbapenems was between 2.4% and 2.7% for the three included (MEM, IPM, and ETP), and for *E. coli*, resistance to IPM was observed in 6.5%. For *A. baumannii,* resistance to MEM was 66.7%, and for FEP it was 57.9%. *P. aeruginosa* had 15.6% and 17.5% resistance to CT and CZA, respectively. For ATM, resistance was 18%; for CAZ, 23.2%; for TZP, 23.4%; and for IPM, 32.9%. *S. maltophilia* exhibited 18.5% resistance to SXT, and 29.2% *S. aureus* strains were MRSA, of which 1% were resistant to LZD. Resistance to VN was not observed.

For bacteria recovered from urine, 46.4% of *E. coli* strains were ESBL producers, 0.9% were resistant to MEM, and 5.4% were resistant to NIT. For *K. pneumoniae,* 47.2% were ESBLs, 0.8% were resistant to MEM, 29.2% were resistant to NIT, and 29.6% were resistant to NOR. For *P. aeruginosa*, resistance to IPM and MEM was 38.5% and 40.1%, respectively; for AK, resistance was 30.58%; for CZA, it was 29.17%; and for TZP, it was 23.5%. For *S. aureus*, 27.458% were MRSA, and no resistance to VN was observed. For *E. faecium*, 41.5% was resistant to VN and 2.4% to LZD.

**Fig 3:**
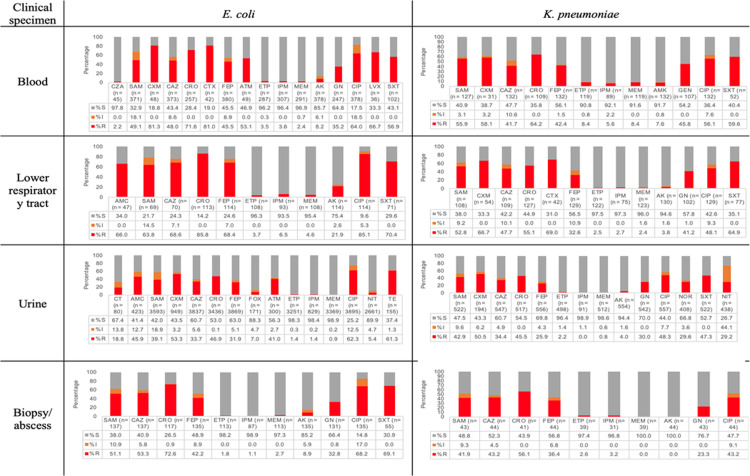
Distribution of antibiotic resistance according to clinical specimens of selected *Enterobacterales: E. coli* (n =**4 618) and** ***K. pneumoniae*** **(n**=883) Amikacin (AK), amoxicillin-clavulanic acid (AMC), aztreonam (ATM), ampicillin-sulbactam (SAM), cefepime (FEP), cefoxitin (FOX), ceftazidime (CAZ), ceftriaxone (CRO), cefuroxime (CXM), ciprofloxacin (CIP), cefotaxime (CTX), ertapenem (ETP), gentamicin (GN), imipenem (IPM), levofloxacin (LVX), meropenem (MEM), nitrofurantoin (NIT), norfloxacin (NOR), tetracycline (TE), trimethoprim-sulfamethoxazole (SXT). S: susceptible; I: intermediate R: resistant.

**Fig 4:**
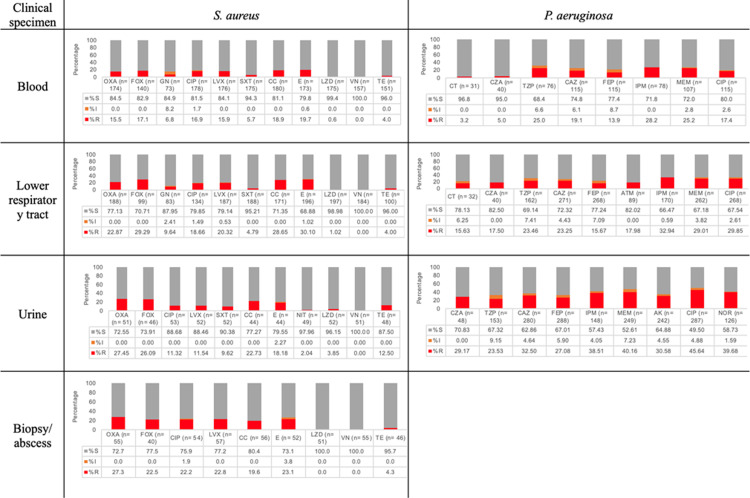
Distribution of antibiotic resistance according to clinical specimens of *S. aureus* (n =**559) and** ***P. aeruginosa*** **(n**=765). Amikacin (AK), aztreonam (ATM), cefepime (FEP), cefoxitin (FOX), ceftazidime (CAZ), ceftazidime-avibactam (CZA), ceftolozane-tazobactam, ceftriaxone (CRO), ciprofloxacin (CIP), clindamycin (CC), erythromycin (E), gentamicin (GN), imipenem (IPM), linezolid (LZD), levofloxacin (LVX), meropenem (MEM), norfloxacin (NOR), oxacillin (OXA), tetracycline (TE), piperacillin-tazobactam (TZP), and vancomycin (VN). S: susceptible; I: intermediate R: resistant.

For clinical isolates recovered from biopsies, abscesses, and aspirates, 68.4% of *E. coli* strains were ESBL producers, 2.7% were resistant to MEM, and 69.1% were resistant to SXT. For *K. pneumoniae*, 50% were ESBL producers. Resistance to ETP, IPM and MEM was observed in 2.6%, 3.2%, and 0%, respectively. For *S. aureus*, 27.3% were categorized as MRSA, and no resistance to VN was reported. In clinical isolates recovered from pleural fluid, 38% of *E. coli* clinical isolates were ESBL producers and resistance to ETP was 8.7%, while resistance to IPM was 9.5%, and resistance to MEM was 7.7%; see Fig 3, Fig 4, and S2 Figure in S1 File.

When comparing according to clinical specimens, there were no differences for *A. baumannii*. For *E. coli*, there was a higher resistance to CIP (*p* < 0.05) and a higher frequency of ESBL producer strains in clinical isolates recovered from LRT (*p* < 0.05).

By contrast, for *K. pneumoniae* the highest resistance to MEM and ETP was observed in blood isolates (*p* < 0.05 for both). *K. pneumoniae* had the highest resistance to CAZ in LRT specimens (*p* < 0.001) and to FEP in blood samples (*p* < 0.001); see S2 Figure in S1 file.

For *P. aeruginosa* from urine samples, a higher resistance to CZA, CAZ, and FEP was more frequently observed when compared to other clinical specimens (*p* < 0.05). Finally, *S. aureus*, MRSA with OXA, and LZD were more frequent in urine (*p* < 0.05), and FOX (surrogate to methicillin) was relevant in LRT (*p* < 0.05); see S2 Table.

### Susceptibility patterns depending on the site of care

Regarding isolates recovered from patients in the EME, 55.2% of *E. coli* were ESBL producers, and resistance to carbapenems was documented in 0.6% for IPM, 0.9% for MEM, and 1.3% for ETP. For *K. pneumoniae,* 42.7% were ESBL producers and 0.8% were resistant to ETP. For *P. aeruginosa,* 22.1% were resistant to MEM and 23.7% were resistant to TZP. For *S. aureus,* 20% were MRSA (both OXA and FOX), and no resistance was reported to VN or LZD.

For clinical isolates recovered for patients in the ICU, 72.9% of *E. coli* strains were ESBL producers, 12.7% were resistant to IPM, and 69.8% were resistant to SXT. For *K. pneumoniae,* 49.1% were ESBL producers; resistance to ETP and MEM was 5%, and for IPM it was 6.7%. For *A. baumannii*, resistance to MEM and IPM was observed at 78%, whereas for *P. aeruginosa,* resistance to IPM was 57.4%, and for TZP it was 49.1%. For *S. aureus,* 26% were MRSA and 1.7% were resistant to LZD.

For clinical isolates recovered from INX hospitalized patients, 59% of *E. coli* strains were ESBL producers, and 3.8% were resistant to IPM. F*or K. pneumoniae,* 59.4% of clinical isolates were ESBL producers, 3.4% were resistant to MEM, and 4.1% were resistant to IPM. In *A. baumannii,* 66.9% had resistance to MEM; for *P. aeruginosa,* 13.5% were resistant to CZA, 24.6% to TZP, and 35.8% to MEM. For *S. maltophilia*, 21.1% of isolates were resistant to SXT. For *S. aureus,* resistance to OXA was documented in 24.6% and to LZD in 0.2%. Resistance to VN was not observed. For *E. faecium,* VN resistance was observed in 37.2% while LZD resistance was in 1.5%.

**Fig 5:**
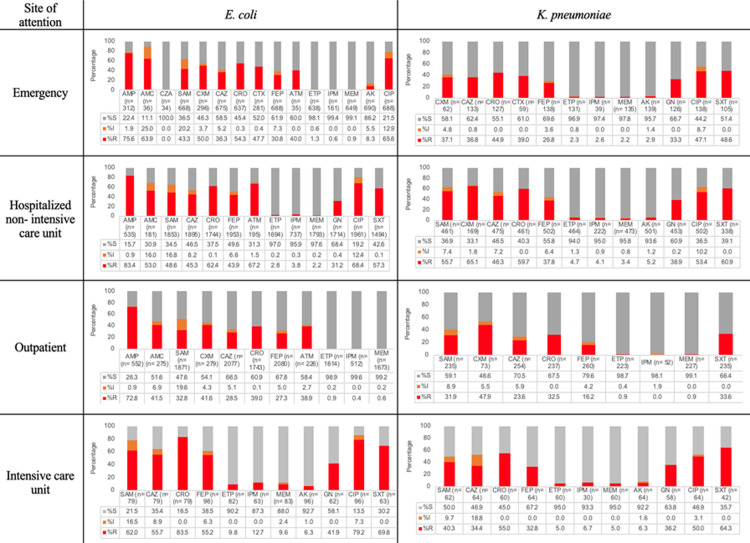
Distribution of antibiotic resistance according to the clinical ward of selected *Enterobacterales.* *E coli* (n= 4 618) and *K. pneumoniae* (n=883). Amikacin (AK), amoxicillin-clavulanic acid (AMC), ampicillin (AMP), ampicillin-sulbactam (SAM), aztreonam (ATM), cefepime (FEP), cefotaxime (CTX), ceftazidime (CAZ), ceftazidime-avibactam (CZA), ceftriaxone (CRO), cefuroxime (CXM), ciprofloxacin (CIP), ertapenem (ETP), gentamicin (GN), imipenem (IPM), meropenem (MEM), trimethoprim-sulfamethoxazole (SXT), S: susceptible; I: intermediate; R: resistant.

**Fig 6:**
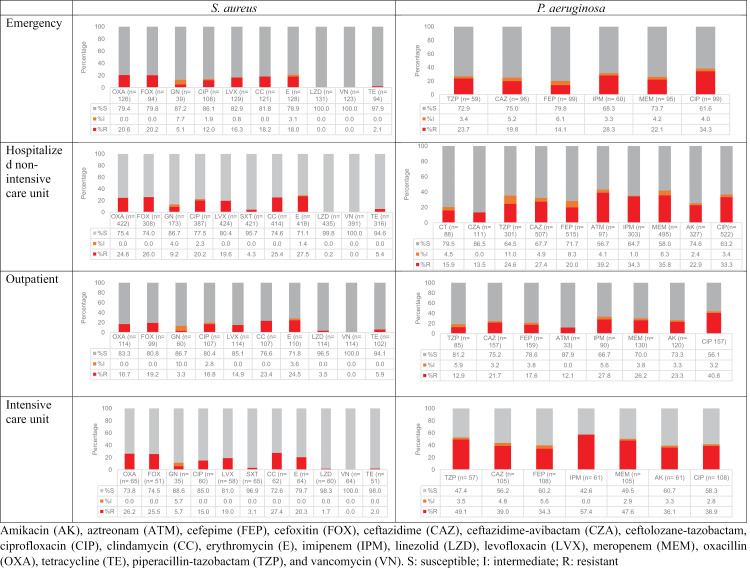
Distribution of antibiotic resistance according to clinical ward of *S. aureus* and *P. aeruginosa*. *S. aureus* (n =**559) and** ***P. aeruginosa*** **(n**=765). Amikacin (AK), aztreonam (ATM), cefepime (FEP), cefoxitin (FOX), ceftazidime (CAZ), ceftazidime-avibactam (CZA), ceftolozane-tazobactam, ciprofloxacin (CIP), clindamycin (CC), erythromycin (E), imipenem (IPM), linezolid (LZD), levofloxacin (LVX), meropenem (MEM), oxacillin (OXA), tetracycline (TE), piperacillin-tazobactam (TZP), and vancomycin (VN). S: susceptible; I: intermediate; R: resistant.

For clinical isolates recovered from outpatients, 38.1% of *E. coli* strains were ESBL producers; 0.6% were resistant to MEM and 38.9% were resistant to ATM. For *K. pneumoniae,* 35.9% were ESBL producers and 0.9% were resistant to MEM. For *P. aeruginosa*, 26.2% were resistant to MEM and 12.9% were resistant to TZP. For *S. aureus,* 19.2% of strains were resistant to FOX and 3.5% to LZD. No VN resistance was observed for *S. aureus;* see Fig 5, Fig 6, and S3 Figure in S1 File*.*

No differences in resistance were observed for *A. baumannii* between groups (S3 Table). The highest frequency of *E. coli* ESBL producers was detected in the INX (*p <* 0.05), and the highest resistance to cephalosporins (*p* < 0.01) and carbapenems (*p* < 0.01) was observed for clinical isolates recovered from patients in the ICU.

For *K. pneumoniae,* the highest proportion of ESBL producer strains (*p* < 0.05) was observed in clinical isolates recovered from the INX area as well as the highest resistance to ETP (*p* < 0.05).

For *P. aeruginosa,* the highest resistance was observed for CAZ, FEP, TZP, IPM, and MEM in clinical isolates recovered from patients in the ICU (*p* < 0.01); see S3 Table.

### Carbapenemases encoding genes

For carbapenemases encoding genes detection, twenty-seven clinical isolates of *E. coli*, five of *K. pneumoniae,* fifty-nine of *A. baumannii,* and fifty-eight of *P. aeruginosa* were received in the six-month collection period*.* All these clinical isolates were non-susceptible to carbapenems.

Regarding non-susceptible to MEM *E. coli*, 16/27 (59.2%) were carriers of *bla*_*NDM,*_ and the *bla*_*KPC*_ gene was detected in 2/5 (40%) strains of *K. pneumoniae* (Table 2)*.* The *bla*_*OXA24*_ gene was detected in 51/59 (89.4%) of *A. baumannii* clinical isolates. For *P. aeruginosa,* the most frequently detected carbapenemase encoding gene was *bla*_*GES*_ (n = 25, 43.1%); see Table 2.

**Table 2:** Distribution of genes associated with carbapenem resistance in gram negatives.

n	Specie	MEM	*bla* *_NDM_*	bla_*KPC*_	*bla* *_VIM_*	*bla* *_IMP_*	*bla* *_GES_*	bla_*OXA48*_	*bla* *_OXA23_*	*bla* *_OXA24_*
13	*E. coli*	R	Pos	Neg	Neg	Neg	ND	Neg	ND	ND
8	*E. coli*	R	Neg	Neg	Neg	Neg	ND	Neg	ND	ND
2	*E. coli*	I	Neg	Neg	Pos	Neg	ND	Neg	ND	ND
3	*E. coli*	R	Pos	Neg	Neg	Neg	ND	Pos	ND	ND
1	*E. coli*	R	Neg	Pos	Neg	Neg	ND	Neg	ND	ND
2	*K. pneumoniae*	R	Neg	Pos	Neg	Neg	ND	Neg	ND	ND
2	*K. pneumoniae*	I	Neg	Neg	Neg	Neg	ND	Pos	ND	ND
1	*K. pneumoniae*	R	Neg	Neg	Neg	Neg	ND	Neg	ND	ND
51	*A.baumannii*	R	ND	ND	ND	ND	ND	ND	Neg	Pos
6	*A. baumannii*	R	ND	ND	ND	ND	ND	ND	Neg	Neg
2	*A. baumannii*	R	ND	ND	ND	ND	ND	ND	Pos	Neg
19	*P. aeruginosa*	R	ND	ND	Neg	Neg	Pos	ND	ND	ND
16	*P. aeruginosa*	R	ND	ND	Neg	Neg	Neg	ND	ND	ND
6	*P. aeruginosa*	R	ND	ND	Pos	Neg	Neg	ND	ND	ND
9	*P. aeruginosa*	R	ND	ND	Neg	Pos	Neg	ND	ND	ND
5	*P. aeruginosa*	R	ND	ND	Neg	Pos	Pos	ND	ND	ND
2	*P. aeruginosa*	R	ND	ND	Pos	Pos	Neg	ND	ND	ND
1	*P. aeruginosa*	R	ND	ND	Pos	Neg	Pos	ND	ND	ND

Pos: Positive, Neg: negative, ND: Not determined, MEM: meropenem.

### MDR, XDR, and PDR strains

MDR, possible XDR, XDR, and possible PDR were identified using the AST results. The bacteria with the most isolates defined as possible PDR was *A. baumannii* (53.9%); meanwhile, *E. coli* had the main strains defined as MDR (Table 3).

**Table 3:** Distribution of MDR, possible XDR, XDR, and possible PDR among species studied.

	MDR n (%)	Possible XDR n (%)	XDR	Possible PDR	Total
*E. coli*	2924 (53.8)	1098 (20.2)	0.0 (0)	13 (0.2)	5438
*K. pneumoniae*	505 (46.9)	366 (34.0)	0.0 (0)	11 (1.0)	1076
*A. baumannii*	165 (67.9)	160 (65.8)	0.0 (0)	131 (53.9)	243
*P. aeruginosa*	267 (27.5)	218 (22.4)	38 (3.9)	169 (17.4)	972

MDR: multidrug-resistant, XDR: extensively drug-resistant, PDR: pan drug-resistant.

## Discussion

Antimicrobial drug resistance is a dynamic and increasing worldwide health concern. In 2019, the WHO reported a list of the ten pathogen threats to global health regarding antimicrobial resistance [20], some of which were included in our work. We also included *S. maltophilia,* which has been scarcely reported in Mexico, and *C. albicans,* which was listed by WHO as a fungal priority pathogen in 2022 [4].

The most frequent microorganism reported was *E. coli*. This bacterial species plays an important role as an etiological agent of infection and was reported as the most frequent cause of death (nearly 800,000 deaths) associated with drug resistance and nearly 300,000 deaths attributable to resistance around the world [3]*.*

Together, *E. coli* and *K. pneumoniae* had a high frequency of strains of ESBL producers in the ≥ 60 years group, representing nearly half for *K. pneumoniae* and approximately 60% for *E. coli.* These findings are similar to those reported in Mexico in 2023, with close to 40% of *E. coli* isolates being reported as ESBL producers [21]. This frequency increased when studied according to clinical specimens, with frequencies higher than 50% in most clinical samples excluding urine. Additionally, ESBL producers were isolated from outpatients (OUT), which may represent the ß-lactamases strains circulating in the community. Unfortunately, we have no data on these outpatients regarding previous hospitalizations. This finding is comparable with the report produced by the Global Antimicrobial Resistance and Use Surveillance System, in which ESBL presence was near 40% for both *E. coli* and *K. pneumoniae* [1]*.*

The selection of the appropriate antimicrobial prescription and usage to prescribe and use should be determined by local epidemiology [22]. Some authors recommend the use of an antimicrobial agent empirically if the frequency of resistance is less than 10% [23]; as relevant results, in our work, *P. aeruginosa* had resistance to CZA (29.1%), TZP (23.5%), IPM (38.5%) and MEM (40.1%); which may suggest that these antibiotics should not be lightly used. Additionally, according to the selection of antibiotics recommended by the Infectious Diseases Society of America in 2024, the preferred antibiotics (defined according to resistance) for the treatment of infections by *P. aeruginosa*, are TZP, CAZ, FEP, ATM, and carbapenems [24]. Once AST results are available, alternative approaches are recommended, such as CT, CZA, and imipenem-relebactam or cefiderocol; thus, our results underline the need for permanent and active surveillance in specific regions, the need for access to existing antimicrobials, and the need for research on the development of new antimicrobial agents is warranted [25].

Regarding the sites of attention, isolates of *E. coli, K. pneumoniae,* and *P. aeruginosa* recovered from samples from patients in the ICU had the highest frequency of resistance to carbapenems. Because of our specific objectives, we did not evaluate specific risk factors according to healthcare areas, but carbapenem-resistance in *Enterobacterales* in patients in the ICUs has been associated with previous emergency stay (> 2 days) and a previous carbapenem administration (*p* < 0.05), among other factors [13,26,27].

The characteristics of specific antibiotic resistance mechanisms play an important role in the proper selection and activity of antibiotics (e.g. depending on the types of carbapenemases) [28]. In this study, 59.2% of *E. coli*, strains were carriers of *bla*_*NDM-1.*_ This result contrasts with our previous 2023 report, in which 84% of carbapenem-resistant clinical isolates of *E. coli* were detected to harbor *bla_NDM_*_*-1*_ (84%). This renders NDM the most frequently circulating carbapenemase among *Enterobacterales*, and an important challenge for the treatment of infections caused by organisms carrying this gene, particularly because there are limited therapeutic options since aztreonam is not available in Mexico [18].

In this study, the highest ranges of resistance to carbapenem were observed in clinical isolates from pleural fluid (7.6% and 9.5%, for MEM and IPM, respectively). In a previous study from the INVIFAR network, 4% and 7% for MEM and IPM resistance, respectively, were reported in a period from 2019 to 2020 [13].

For *S. maltophilia* in LRT, a resistance of 18.4% was observed for SXT. Different frequencies of SXT resistance have been reported according to the age of patients and their clinical condition. In an outbreak of *S. maltophilia* at the Hospital Infantil de Mexico, a tertiary care pediatric hospital, resistance to SXT was reported to be as high as 76.6% and in contrast, in strains recovered over 16 years from an oncologic population, the susceptibility was 95% and in clinical isolates recovered from orthopedic patients the susceptibility was 99% [29]*.*

Recognizing the national situation regarding drug resistance is the first step for the development and implementation of local guidelines for antibiotic use. Some countries and regions have implemented antimicrobial stewardship guidelines for the correct use of these antibiotics at regional or national levels [30–32]. In our work, the voluntary and cooperative participation of various centers around the country has provided valuable information about the antibiotic resistance of relevant bacterial species, which prompts our network to continue the active surveillance to provide local data that may be useful for antibiotic prescriptions in Mexico.

Finally, *C. albicans* recovered from blood had a resistance to VOR of 2.4%. Resistance to VOR was reported in Paraguayan hospitals with percentages of resistance similar to ours (2.5%) [33]. Data from our country is limited for fungi, and in this study, few hospitals provided data related to *C. albicans.* In our region, few centers can perform AST of yeast and molds. The promotion through the INVIFAR network of capacitation in mycology will open a new branch to make surveillance possible. It is important to mention that none of *C. albicans* included showed resistance to caspofungin, a family member of the equinocandins drug group, even in isolates recovered from blood cultures.

To improve the reliability of results, the INVIFAR network is working to support microbiological procedures and results by an annual quality control exercise, in which the INVIFAR centers participate. Additionally, some centers have external control systems.

Our work has some limitations. First, the participating centers used different methodologies or instruments to perform the AST. Second, some antibiotics, such as tigecycline and colistin, were not included: the first because there are no breakpoints to interpretation, and the second because almost all centers use colistin from semi-automatized systems with no reliable results. Third, clinical data, including antibiotics administrated, were not available for this study and finally, antimicrobials with n < 30 were not considered as recommendations.

In conclusion, carbapenem resistance frequencies were higher for *E. coli* in the 0–17 years group and for *K. pneumoniae*, *P. aeruginosa,* and *A. baumannii* in the 18–59 years group. *K. pneumoniae* had the highest resistance to carbapenems in blood isolates and patients attended in the INX area. *E. coli* and *P. aeruginosa* had the highest carbapenem resistance in the ICU. A high MDR was observed for *A. baumannii* and *E. coli* strains. A high susceptibility to caspofungin and voriconazole was observed for *C. albicans* collected from blood.

## Supporting information

S1 File Distribution of antibiotic resistance according from other species/age groups. *S. maltophilia* (n= 105), *S. aureus* (n= 559), *E. faecium* (n= 331). (DOCX)

S1 Table P values for comparison among age groups. (DOCX)

S2 Table P values for comparisons among clinical specimens. (DOCX)

S3 Table P value for comparison among clinical wards. (DOCX)
