# Supplementary material for: The ongoing antibiotic resistance and carbapenemase encoding genotypes surveillance. The first quarter report of the INVIFAR network for 2024
Source: PLoS One. 2025 Apr 16;20(4):e0319441. doi: 10.1371/journal.pone.0319441 (PMC12002462; doi:10.1371/journal.pone.0319441)
Supplement: S1 File — (DOCX) [file pone.0319441.s001.docx]

Suppl Fig 1. Distribution of antibiotic resistance according from other species/age groups. *S. maltophilia* (n= 105), *S. aureus* (n= 559), *E. faecium* (n= 331).

|  | Other species and age range | |
| --- | --- | --- |
| *S. maltophilia*/0-17 years | *S. maltophilia*/≥60 years | *S. aureus/*0-17 years |
|  |  |  |
| *E. faecium*/ ≥60 years | | *S. aureus*/ ≥60 years |
|  | |  |

Ampicillin (AMP), cefoxitin (FOX), ciprofloxacin (CIP), clindamycin (CC), erythromycin (E), gentamicin (GN), gentamicin high levels (GNH), linezolid (LZD), levofloxacin (LVX), oxacillin (OXA), streptomycin high levels (STRH), tetracycline (TE), trimethoprim-sulfamethoxazole (SXT), and vancomycin (VN). S: susceptible; I: intermedio; R: resistant

Suppl. Fig 2. Distribution of antibiotic resistance according to clinical specimen of selected species. *S. maltophilia* (m= 105), *A. baumannii* (n= 182), *E. faecium* (n= 221), *E. coli* (n= 4618), *C. albicans* (n= 42).

| *S. maltophilia*/respiratory | *A. baumannii*/respiratory | *C.albicans/*blood |
| --- | --- | --- |
|  |  |  |
| 1. *baumannii*/blood | *E. faecium*/urine | *E. coli*/pleural |
|  |  |  |

Amikacin (AK), ampicillin (AMP), ampicillin-sulbactam (SAM), caspofungin (CAS), cefepime (FEP), ceftriaxone (CRO), ciprofloxacin (CIP), erythromycin (E), ertapenem (ETP), gentamicin (GN), gentamicin high levels (GNH), imipenem (IPM), linezolid (LZD), levofloxacin (LVX), meropenem (MEM), nitrofurantoin (NIT), streptomycin high levels (STRH), tetracycline (TE), trimethoprim-sulfamethoxazole (SXT), vancomycin (VN) and voriconazole (VOR). S: susceptible; I: intermediate; R: resistant.

Suppl. Fig 3. Distribution of antibiotic resistance of other species according to clinical ward. *S. maltophilia* (n= 105), *E. faecium* (n= 331), *A. baumannii* (n= 182).

| *S. maltophilia/*hospitalized non-ICU | *E. faecium/*hospitalized non ICU |
| --- | --- |
|  |  |
| *A. baumannii*/hospitalized-non ICU | *A. baumannii*/ICU |
|  |  |

Amikacin (AK), ampicillin (AMP), ampicillin-sulbactam (SAM), ciprofloxacin (CIP), erythromycin (E), gentamicin (GN), gentamicin high levels (GNH), imipenem (IPM), linezolid (LZD), levofloxacin (LVX), meropenem (MEM), penicillin (P), streptomycin high levels (STRH), trimethoprim-sulfamethoxazole (SXT), and vancomycin (VN). S: susceptible; I: intermediate; R: resistant
