# Supplementary material for: The ongoing antibiotic resistance and carbapenemase encoding genotypes surveillance. The first quarter report of the INVIFAR network for 2024
Source: PLoS One. 2025 Apr 16;20(4):e0319441. doi: 10.1371/journal.pone.0319441 (PMC12002462; doi:10.1371/journal.pone.0319441)
Supplement: S1 Table — (DOCX) [file pone.0319441.s002.docx]

Suppl. Table 1. P values for comparison among age groups

| *E. coli* | | | | |
| --- | --- | --- | --- | --- |
| Antibiotic/ESBL | Age group (years) | Susceptible | Non-susceptible | p (chi^2^) |
|  |  |  |  |  |
| ESBL | 0-17 | 130 | 123 | 0.03 |
| ESBL | 18-59 | 852 | 788 |  |
| ESBL | >60 | 836 | 924 |  |
| SAM | 0-17 | 124 | 213 | 0.004 |
| SAM | 18-59 | 950 | 1281 |  |
| SAM | >60 | 811 | 1324 |  |
| CAZ | 0-17 | 216 | 143 | 0.01 |
| CAZ | 18-59 | 1402 | 955 |  |
| CAZ | >60 | 1241 | 1003 |  |
| CRO | 0-17 | 186 | 156 | 0.002 |
| CRO | 18-59 | 1029 | 1038 |  |
| CRO | >60 | 929 | 1103 |  |
| FEP | 0-17 | 229 | 133 | 0.008 |
| FEP | 18-59 | 1465 | 924 |  |
| FEP | >60 | 1318 | 980 |  |
| ATM | 0-17 | 21 | 19 | 0.592 |
| ATM | 18-59 | 105 | 118 |  |
| ATM | >60 | 91 | 115 |  |
| ETP | 0-17 | 302 | 8 | 0.477 |
| ETP | 18-59 | 1909 | 47 |  |
| ETP | >60 | 1972 | 38 |  |
| IPM | 0-17 | 103 | 2 | 0.048 |
| IPM | 18-59 | 692 | 28 |  |
| IPM | >60 | 664 | 12 |  |
| MEM | 0-17 | 318 | 3 | 0.105 |
| MEM | 18-59 | 1975 | 41 |  |
| MEM | >60 | 2054 | 27 |  |

ESBL: extended spectrum β-lactamase, SAM: ampicillin-sulbactam, CAZ: ceftazidime, CRO: ceftriaxone, FEP: cefepime, ATM: aztreonam, ETP: ertapenem, IPM: imipenem, MEM: meropenem.

| *K. pneumoniae* | | | | |
| --- | --- | --- | --- | --- |
| Antibiotic/ESBL | Age group (years) | Susceptible | Non-susceptible | p (chi^2^) |
| ESBL | 0-17 | 39 | 60 | 0.055 |
| ESBL | 18-59 | 196 | 174 |  |
| ESBL | >60 | 170 | 174 |  |
| SAM | 0-17 | 44 | 72 | 0.16 |
| SAM | 18-59 | 200 | 232 |  |
| SAM | >60 | 188 | 204 |  |
| CAZ | 0-17 | 53 | 65 | 0.047 |
| CAZ | 18-59 | 258 | 193 |  |
| CAZ | >60 | 230 | 176 |  |
| CRO | 0-17 | 39 | 69 | 0.013 |
| CRO | 18-59 | 221 | 212 |  |
| CRO | >60 | 202 | 192 |  |
| FEP | 0-17 | 64 | 53 | 0.041 |
| FEP | 18-59 | 312 | 159 |  |
| FEP | >60 | 284 | 141 |  |
| ETP | 0-17 | 101 | 7 | 0.324 |
| ETP | 18-59 | 407 | 18 |  |
| ETP | >60 | 383 | 13 |  |
| IPM | 0-17 | 32 | 1 | 0.017 |
| IPM | 18-59 | 168 | 12 |  |
| IPM | >60 | 151 | 1 |  |
| MEM | 0-17 | 105 | 5 | 0.065 |
| MEM | 18-59 | 415 | 17 |  |
| MEM | >60 | 398 | 6 |  |
| GN | 0-17 | 65 | 44 | 0.117 |
| GN | 18-59 | 299 | 129 |  |
| GN | >60 | 262 | 132 |  |
| CIP | 0-17 | 48 | 70 | 0.712 |
| CIP | 18-59 | 208 | 263 |  |
| CIP | >60 | 179 | 247 |  |
| SXT | 0-17 | 36 | 60 | 0.041 |
| SXT | 18-59 | 177 | 163 |  |
| SXT | >60 | 164 | 166 |  |

ESBL: extended spectrum β-lactamase, SAM: ampicillin-sulbactam, CAZ: ceftazidime, CRO: ceftriaxone, FEP: cefepime, ETP: ertapenem, IPM: imipenem, MEM: meropenem, GN: gentamicin, CIP: ciprofloxacin, SXT: trimetoprim-sulfamethoxazole

| *Acinetobacter baumannii* | | | | | |
| --- | --- | --- | --- | --- | --- |
| Antibiotic | Age group (years) | Susceptible | Non-susceptible | p (chi^2^) | Fisher exact test |
| SAM | 0-17 | 19 | 13 | 0.0001 | na |
| SAM | 18-59 | 29 | 106 |  |  |
| SAM | >60 | 21 | 38 |  |  |
| IPM | 18-59 | 12 | 68 | na | 0.5832 |
| IPM | >60 | 7 | 27 |  |  |
| MEM | 0-17 | 19 | 13 | 0.00021 | na |
| MEM | 18-59 | 30 | 104 |  |  |
| MEM | >60 | 18 | 37 |  |  |
| AK | 18-59 | 23 | 59 | na | 0.5068 |
| AK | >60 | 12 | 22 |  |  |
| GN | 18-59 | 26 | 63 | na | 0.4542 |
| GN | >60 | 19 | 33 |  |  |
| CIP | 0-17 | 19 | 13 | 0.00001 | na |
| CIP | 18-59 | 24 | 110 |  |  |
| CIP | >60 | 15 | 42 |  |  |

SAM: ampicillin-sulbactam, IPM: imipenem, MEM: meropenem, AK: amikacin, GN: gentamicin, CIP: ciprofloxacin, na: not apply

| *P. aeruginosa* | | | | |
| --- | --- | --- | --- | --- |
| **Antibiotic** | Age group (years) | Susceptible | Non-susceptible | p (chi^2^) |
| CZA | 0-17 | 39 | 1 | 0.012 |
| CZA | 18-59 | 57 | 17 |  |
| CZA | >60 | 42 | 13 |  |
| CT | 0-17 | 41 | 1 | 0.00025 |
| CT | 18-59 | 29 | 13 |  |
| CT | >60 | 20 | 13 |  |
| TZP | 0-17 | 54 | 23 | 0.362 |
| TZP | 18-59 | 175 | 92 |  |
| TZP | >60 | 127 | 50 |  |
| CAZ | 0-17 | 80 | 25 | 0.169 |
| CAZ | 18-59 | 328 | 152 |  |
| CAZ | >60 | 209 | 106 |  |
| FEP | 0-17 | 90 | 16 | 0.01 |
| FEP | 18-59 | 349 | 137 |  |
| FEP | >60 | 227 | 97 |  |
| IPM | 0-17 | 57 | 15 | 0.013 |
| IPM | 18-59 | 163 | 107 |  |
| IPM | >60 | 117 | 66 |  |
| MEM | 0-17 | 79 | 24 | 0.004 |
| MEM | 18-59 | 272 | 188 |  |
| MEM | >60 | 184 | 114 |  |
| CIP | 0-17 | 89 | 16 | 0.0000012 |
| CIP | 18-59 | 303 | 187 |  |
| CIP | >60 | 183 | 141 |  |

CZA: ceftazidime-avibactam, CT: ceftolozane-tazobactam, TZP: piperacillin-tazobactam, CAZ: ceftazidime, FEP: cefepime, IPM: imipenem, MEM: meropenem, CIP: ciprofloxacin.

| *S. maltophilia* | | | | |
| --- | --- | --- | --- | --- |
| Antibiotic | Age group (years) | Susceptible | Non-susceptible | p (chi^2^) |
| SXT | 0-17 | 30 | 2 | 0.088 |
| SXT | 18-59 | 55 | 18 |  |
| SXT | >60 | 30 | 8 |  |

SXT: trimethoprim-sulfamethoxazole, na: not apply

| *S. aureus* | | | | |
| --- | --- | --- | --- | --- |
| **Antibiotic** | Age group (years) | Susceptible | Non-susceptible | p (chi^2^) |
| GN | 0-17 | 58 | 12 | 0.378 |
| GN | 18-59 | 166 | 23 |  |
| GN | >60 | 74 | 8 |  |
| CIP | 0-17 | 100 | 5 | 0.00001 |
| CIP | 18-59 | 312 | 84 |  |
| CIP | >60 | 141 | 54 |  |
| LVX | 0-17 | 100 | 3 | 0.00004 |
| LVX | 18-59 | 344 | 91 |  |
| LVX | >60 | 171 | 50 |  |
| CC | 0-17 | 95 | 14 | 0.008 |
| CC | 18-59 | 309 | 111 |  |
| CC | >60 | 153 | 57 |  |
| E | 0-17 | 93 | 18 | 0.001 |
| E | 18-59 | 319 | 111 |  |
| E | >60 | 140 | 74 |  |
| LZD | 0-17 | 104 | 1 | >0.05 |
| LZD | 18-59 | 427 | 3 |  |
| LZD | >60 | 219 | 2 |  |
| VN | 0-17 | 100 | 0 | na |
| VN | 18-59 | 408 | 0 |  |
| VN | >60 | 220 | 0 |  |
|  |  |  |  |  |

GN: gentamicin, CIP: ciprofloxacin, LVX: levofloxacin, CC: clindamycin, E: erythromycin, LZD: linezolid, VN: vancomycin

| *E. faecium* | | | | | |
| --- | --- | --- | --- | --- | --- |
| **Antibiotic** | age group (years) | Susceptible | Non-susceptible | p (chi^2^) | Fisher exact test |
| AMP | 18-59 | 27 | 80 | na | 0.197 |
| AMP | >60 | 12 | 62 |  |  |
| GNH | 18-59 | 80 | 6 | na | 0.467 |
| GNH | >60 | 62 | 2 |  |  |
| STRH | 18-59 | 75 | 9 | na | 0.777 |
| STRH | >60 | 58 | 5 |  |  |
| CIP | 18-59 | 75 | 9 | na | 0.0001 |
| CIP | >60 | 10 | 63 |  |  |
| LVX | 18-59 | 40 | 63 | na | 0.072 |
| LVX | >60 | 18 | 54 |  |  |
| E | 18-59 | 5 | 92 | na | 0.741 |
| E | >60 | 5 | 62 |  |  |
| LZD | 18-59 | 101 | 3 |  | >0.05 |
| LZD | >60 | 68 | 2 | na |  |
| VN | 18-59 | 59 | 40 |  | 0.521 |
| VN | >60 | 44 | 24 | na |  |
| TE | 18-59 | 35 | 41 | na |  |
| TE | >60 | 33 | 21 |  | 0.109 |

AMP: ampicillin, GNH; gentamicin high, STRH; streptomycin high, CIP: ciprofloxacin, LVX: levofloxacin: E: erythromycin, LZD: linezolid, VN: vancomycin, TE: tetracycline, na: not apply
