# Supplementary material for: The ongoing antibiotic resistance and carbapenemase encoding genotypes surveillance. The first quarter report of the INVIFAR network for 2024
Source: PLoS One. 2025 Apr 16;20(4):e0319441. doi: 10.1371/journal.pone.0319441 (PMC12002462; doi:10.1371/journal.pone.0319441)
Supplement: S2 Table — (DOCX) [file pone.0319441.s003.docx]

Suppl table 2. P values for comparisons among clinical specimens

| *E. coli* | | | | | | |
| --- | --- | --- | --- | --- | --- | --- |
| Antibiotic | Clinical specimen | Susceptible | Non-susceptible | p (chi^2^) | Fisher exact test | Excluding PF |
| ESBL | Blood | 73 | 183 | <0.01 | na | <0.01 |
| ESBL | BX/ABS | 31 | 67 |  |  |  |
| ESBL | LRT | 10 | 46 |  |  |  |
| ESBL | Urine | 1499 | 1297 |  |  |  |
| ESBL | PF | 7 | 15 |  |  |  |
| SAM | Blood | 122 | 249 | <0.01 | na | <0.01 |
| SAM | BX/ABS | 52 | 85 |  |  |  |
| SAM | LRT | 15 | 54 |  |  |  |
| SAM | Urine | 1508 | 2085 |  |  |  |
| SAM | PF | 9 | 17 |  |  |  |
| CAZ | Blood | 162 | 211 | <0.01 | na | <0.01 |
| CAZ | BX/ABS | 56 | 81 |  |  |  |
| CAZ | LRT | 17 | 53 |  |  |  |
| CAZ | Urine | 2329 | 1508 |  |  |  |
| CAZ | PF | NA | NA |  |  |  |
| CRO | Blood | 73 | 184 | <0.01 | na | <0.01 |
| CRO | BX/ABS | 31 | 86 |  |  |  |
| CRO | LRT | 16 | 97 |  |  |  |
| CRO | Urine | 1821 | 1615 |  |  |  |
| CRO | PF | 2 | 16 |  |  |  |
| FEP | Blood | 173 | 207 | <0.01 | na | <0.01 |
| FEP | BX/ABS | 66 | 69 |  |  |  |
| FEP | LRT | 28 | 86 |  |  |  |
| FEP | Urine | 2437 | 1432 |  |  |  |
| FEP | PF | 5 | 23 |  |  |  |
| ETP | Blood | 276 | 11 | >0.05 | na | 0.046 |
| ETP | BX/ABS | 111 | 2 |  |  |  |
| ETP | LRT | 104 | 4 |  |  |  |
| ETP | Urine | 3195 | 56 |  |  |  |
| ETP | PF | 21 | 2 |  |  |  |
| IPM | Blood | 296 | 11 | >0.05 | na | 0.009 |
| IPM | BX/ABS | 86 | 1 |  |  |  |
| IPM | LRT | 87 | 6 |  |  |  |
| IPM | Urine | 816 | 13 |  |  |  |
| IPM | PF | 19 | 2 |  |  |  |
| MEM | Blood | 282 | 9 | >0.5 | na | 0.001 |
| MEM | BX/ABS | 110 | 3 |  |  |  |
| MEM | LRT | 103 | 5 |  |  |  |
| MEM | Urine | 3331 | 38 |  |  |  |
| MEM | PF | 24 | 2 |  |  |  |
| CIP | Blood | 66 | 312 | <0.01 | na | <0.01 |
| CIP | BX/ABS | 20 | 115 |  |  |  |
| CIP | LRT | 11 | 103 |  |  |  |
| CIP | Urine | 982 | 2913 |  |  |  |
| CIP | PF | 3 | 25 |  |  |  |
| SXT | Blood | 44 | 58 | 0.126 | na | 0.126 |
| SXT | BX/ABS | 17 | 38 |  |  |  |
| SXT | LRT | 21 | 50 |  |  |  |
| SXT | Urine | NA | NA |  |  |  |
| SXT | PF | NA | NA |  |  |  |

ESBL: extended spectrum β-lactamase, SAM: ampicillin-sulbactam, CAZ: ceftazidime, FEP: cefepime, ETP: ertapenem, IPM: imipenem, MEM: meropenem, CIP: ciprofloxacin, SXT: trimetoprim-sulfamethoxazole. BX: biopsy, ABS: abscess, LRT: lower respiratory tract, PF: pleural fluid, NA: not apply, NA: not available

| *K. pneumoniae* | | | | |
| --- | --- | --- | --- | --- |
| Antibiotic | Clinical specimen | Susceptible | Non-susceptible | p (chi^2^) |
| ESBL | Blood | 52 | 57 | 0.133 |
| ESBL | BX/ABS | 19 | 19 |  |
| ESBL | LRT | 36 | 55 |  |
| ESBL | Urine | 235 | 210 |  |
| CAZ | Blood | 63 | 69 | 0.001 |
| CAZ | BX/ABS | 23 | 21 |  |
| CAZ | LRT | 46 | 63 |  |
| CAZ | Urine | 332 | 215 |  |
| CRO | Blood | 39 | 70 | 0.002 |
| CRO | BX/ABS | 18 | 23 |  |
| CRO | LRT | 57 | 70 |  |
| CRO | Urine | 282 | 235 |  |
| FEP | Blood | 74 | 58 | 0.001 |
| FEP | BX/ABS | 25 | 19 |  |
| FEP | LRT | 73 | 56 |  |
| FEP | Urine | 388 | 168 |  |
| ETP | Blood | 108 | 11 | 0.029 |
| ETP | BX/ABS | 38 | 1 |  |
| ETP | LRT | 119 | 3 |  |
| ETP | Urine | 480 | 18 |  |
| IPM | Blood | 82 | 7 | 0.109 |
| IPM | BX/ABS | 30 | 1 |  |
| IPM | LRT | 73 | 2 |  |
| IPM | Urine | 90 | 1 |  |
| MEM | Blood | 109 | 10 | <0.01 |
| MEM | BX/ABS | 39 | 0 |  |
| MEM | LRT | 118 | 5 |  |
| MEM | Urine | 505 | 7 |  |
| SXT | Blood | 21 | 31 | 0.006 |
| SXT | BX/ABS | N | N |  |
| SXT | LRT | 27 | 50 |  |
| SXT | Urine | 275 | 247 |  |

ESBL: extended spectrum β-lactamase, CAZ: ceftazidime, CRO: ceftriaxone, FEP: cefepime, ETP: ertapenem, IPM: imipenem, MEM: meropenem, SXT: trimetoprim-sulfamethoxazole. BX: biopsy, ABS: abscess, LRT: lower respiratory tract, PF: pleural fluid, NA: not apply,

| *Acinetobacter baumannii* | | | | | |
| --- | --- | --- | --- | --- | --- |
| Antibiotic | Clinical specimen | Susceptible | Non-susceptible | p (chi^2^) | Fisher exact test |
| SAM | Blood | 18 | 44 | na | 0.2574 |
| SAM | LRT | 24 | 37 |  |  |
| FEP | Blood | 17 | 44 |  | 0.5534 |
| FEP | LRT | 19 | 38 |  |  |
| IPM | Blood | 12 | 33 | na | na |
| IPM | LRT | N | N |  |  |
| MEM | Blood | 20 | 41 | na | >0.05 |
| MEM | LRT | 18 | 39 |  |  |
| AK | Blood | 12 | 32 | na | 0.8026 |
| AK | LRT | 11 | 23 |  |  |
| CIP | Blood | 19 | 43 | na | 0.8446 |
| CIP | LRT | 19 | 38 |  |  |
| SXT | Blood | 9 | 27 | na | 0.4261 |
| SXT | LRT | 11 | 20 |  |  |

SAM: ampicillin-sulbactam, FEP: cefepime, IPM: imipenem, MEM: meropenem, AK: amikacin, CIP: ciprofloxacin, SXT: trimetoprim-sulfamethoxazole. BX: biopsy, ABS: abscess, LRT: lower respiratory tract, PF: pleural fluid, NA: not apply.

| *P. aeruginosa* | | | | |
| --- | --- | --- | --- | --- |
| Antibiotic | Clinical specimen | Susceptible | Non-susceptible | p (chi^2^) |
| CZA | Blood | 38 | 2 | 0.013 |
| CZA | LRT | 33 | 7 |  |
| CZA | Urine | 34 | 14 |  |
| CT | Blood | 30 | 1 | 0.026 |
| CT | LRT | 25 | 7 |  |
| CT | Urine | N | N |  |
| TZP | Blood | 52 | 24 | 0.942 |
| TZP | LRT | 112 | 50 |  |
| TZP | Urine | 103 | 50 |  |
| CAZ | Blood | 86 | 29 | 0.018 |
| CAZ | LRT | 196 | 75 |  |
| CAZ | Urine | 176 | 104 |  |
| FEP | Blood | 89 | 26 | 0.012 |
| FEP | LRT | 207 | 61 |  |
| FEP | Urine | 193 | 95 |  |
| IPM | Blood | 56 | 22 | 0.071 |
| IPM | LRT | 113 | 57 |  |
| IPM | Urine | 85 | 63 |  |
| MEM | Blood | 77 | 30 | <0.01 |
| MEM | LRT | 176 | 86 |  |
| MEM | Urine | 131 | 118 |  |
| CIP | Blood | 92 | 23 | <0.01 |
| CIP | LRT | 181 | 87 |  |
| CIP | Urine | 142 | 145 |  |

CZA: ceftazidime-avibactam, CT: ceftolozane-tazobactam, TZP: piperacillin-tazobactam, CAZ: ceftazidime, FEP: cefepime, IPM: imipenem, MEM: meropenem, CIP: ciprofloxacin, LRT: lower respiratory tract.

| *S. aureus* | | | | |
| --- | --- | --- | --- | --- |
| Antibiotic | Clinical specimen | Susceptible | Non-susceptible | p (chi^2^) |
| OXA | Blood | 147 | 27 | 0.107 |
| OXA | BX/ABS | 40 | 15 |  |
| OXA | LRT | 145 | 43 |  |
| OXA | Urine | 37 | 14 |  |
| FOX | Blood | 116 | 24 | 0.157 |
| FOX | BX/ABS | 31 | 9 |  |
| FOX | LRT | 70 | 29 |  |
| FOX | Urine | 34 | 12 |  |
| GN | Blood | 62 | 11 | 0.581 |
| GN | BX/ABS | N | N |  |
| GN | LRT | 73 | 10 |  |
| GN | Urine | N | N |  |
| CIP | Blood | 145 | 33 | 0.379 |
| CIP | BX/ABS | 41 | 13 |  |
| CIP | LRT | 107 | 27 |  |
| CIP | Urine | 47 | 6 |  |
| LVX | Blood | 148 | 28 | 0.276 |
| LVX | BX/ABS | 44 | 13 |  |
| LVX | LRT | 148 | 39 |  |
| LVX | Urine | 46 | 6 |  |
| CC | Blood | 146 | 34 | 0.161 |
| CC | BX/ABS | 45 | 11 |  |
| CC | LRT | 122 | 49 |  |
| CC | Urine | 34 | 10 |  |
| E | Blood | 138 | 35 | 0.093 |
| E | BX/ABS | 38 | 14 |  |
| E | LRT | 135 | 61 |  |
| E | Urine | 35 | 9 |  |
| LZD | Blood | 174 | 1 | >0.05 |
| LZD | BX/ABS | 51 | 0 |  |
| LZD | LRT | 195 | 2 |  |
| LZD | Urine | 50 | 2 |  |
| VN | Blood | 157 | 0 | na |
| VN | BX/ABS | 55 | 0 |  |
| VN | LRT | 184 | 0 |  |
| VN | Urine | 51 | 0 |  |
| TE | Blood | 145 | 6 | 0.112 |
| TE | BX/ABS | 44 | 2 |  |
| TE | LRT | 96 | 4 |  |
| TE | Urine | 42 | 6 |  |

OXA: oxacillin, FOX: cefoxitin, GN: gentamicin, CIP: ciprofloxacin, LVX: levofloxacin, CC: clindamycin, E: erythromycin, LZD: linezolid, VN: vancomycin, TE: tetracycline, BX: biopsy, ABS: abscess, LRT: lower respiratory tract, NA: not apply.
