# Supplementary material for: The ongoing antibiotic resistance and carbapenemase encoding genotypes surveillance. The first quarter report of the INVIFAR network for 2024
Source: PLoS One. 2025 Apr 16;20(4):e0319441. doi: 10.1371/journal.pone.0319441 (PMC12002462; doi:10.1371/journal.pone.0319441)
Supplement: S3 Table — (DOCX) [file pone.0319441.s004.docx]

Suppl table 3. P value for comparison among clinical wards.

| *E. coli* | | | | | |
| --- | --- | --- | --- | --- | --- |
| Antibiotic/ESBL | Localization | Susceptible | Non-susceptible | p (chi^2^) | Fisher exact test |
| ESBL | ICU | 16 | 43 | <0.01 | na |
| ESBL | OUT | 840 | 518 |  |  |
| ESBL | EME | 276 | 340 |  |  |
| ESBL | INX | 84 | 451 |  |  |
| SAM | ICU | 17 | 62 | <0.01 | na |
| SAM | OUT | 890 | 981 |  |  |
| SAM | EME | 244 | 424 |  |  |
| SAM | INX | 639 | 1214 |  |  |
| CAZ | ICU | 28 | 51 | <0.01 | na |
| CAZ | OUT | 1381 | 696 |  |  |
| CAZ | EME | 395 | 280 |  |  |
| CAZ | INX | 882 | 1013 |  |  |
| CRO | ICU | 13 | 66 | <0.01 | na |
| CRO | OUT | 1062 | 681 |  |  |
| CRO | EME | 289 | 348 |  |  |
| CRO | INX | 654 | 1090 |  |  |
| FEP | ICU | 37 | 59 | <0.01 | na |
| FEP | OUT | 1410 | 670 |  |  |
| FEP | EME | 426 | 262 |  |  |
| FEP | INX | 969 | 984 |  |  |
| ETP | ICU | 74 | 8 | <0.01 | na |
| ETP | OUT | 1597 | 17 |  |  |
| ETP | EME | 626 | 12 |  |  |
| ETP | INX | 1643 | 51 |  |  |
| IPM | ICU | 55 | 8 | <0.01 | na |
| IPM | OUT | 510 | 2 |  |  |
| IPM | EME | 160 | 1 |  |  |
| IPM | INX | 707 | 30 |  |  |
| MEM | ICU | 73 | 10 | <0.01 | na |
| MEM | OUT | 1659 | 14 |  |  |
| MEM | EME | 643 | 6 |  |  |
| MEM | INX | 1721 | 42 |  |  |
| CIP | ICU | 13 | 83 | 0.135 | na |
| CIP | OUT | ND | ND |  |  |
| CIP | EME | 148 | 540 |  |  |
| CIP | INX | 377 | 1584 |  |  |
| SXT | ICU | 19 | 44 | na | 0.051 |
| SXT | OUT | ND | ND |  |  |
| SXT | EME | ND | ND |  |  |
| SXT | INX | 636 | 858 |  |  |

ESBL: extended spectrum β-lactamase, SAM: ampicillin-sulbactam, CAZ: ceftazidime, CRO: ceftriaxone, FEP: cefepime, ETP: ertapenem, IPM: imipenem, MEM: meropenem, CIP: ciprofloxacin, SXT: trimetoprim-sulfamethoxazole. ICU: intensive care unit, OUT: outpatients, EME: emergency, INX: hospitalized non-ICU, na: not apply.

| *K. pneumoniae* | | | | |
| --- | --- | --- | --- | --- |
| Antibiotic/ESBL | Localization | Susceptible | Non-susceptible | p (chi^2^) |
| ESBL | ICU | 28 | 27 | <0.01 |
| ESBL | OUT | 127 | 71 |  |
| ESBL | EME | 71 | 53 |  |
| ESBL | INX | 170 | 246 |  |
| CAZ | ICU | 30 | 34 | <0.01 |
| CAZ | OUT | 179 | 75 |  |
| CAZ | EME | 83 | 50 |  |
| CAZ | INX | 221 | 254 |  |
| CRO | ICU | 27 | 33 | <0.01 |
| CRO | OUT | 160 | 77 |  |
| CRO | EME | 70 | 57 |  |
| CRO | INX | 186 | 275 |  |
| FEP | ICU | 43 | 21 | <0.01 |
| FEP | OUT | 207 | 53 |  |
| FEP | EME | 96 | 42 |  |
| FEP | INX | 280 | 222 |  |
| ETP | ICU | 57 | 3 | 0.035 |
| ETP | OUT | 220 | 3 |  |
| ETP | EME | 127 | 4 |  |
| ETP | INX | 436 | 28 |  |
| IPM | ICU | 28 | 2 | 0.657 |
| IPM | OUT | 51 | 1 |  |
| IPM | EME | 38 | 1 |  |
| IPM | INX | 211 | 11 |  |
| MEM | ICU | 57 | 3 | 0.081 |
| MEM | OUT | 225 | 2 |  |
| MEM | EME | 132 | 3 |  |
| MEM | INX | 453 | 20 |  |
| SXT | ICU | 15 | 27 | <0.01 |
| SXT | OUT | 156 | 79 |  |
| SXT | EME | 54 | 51 |  |
| SXT | INX | 132 | 206 |  |

ESBL: extended spectrum β-lactamase, CAZ: ceftazidime, CRO: ceftriaxone, FEP: cefepime, ETP: ertapenem, IPM: imipenem, MEM: meropenem, SXT: trimetoprim-sulfamethoxazole. ICU: intensive care unit, OUT: outpatients, EME: emergency, INX: hospitalized non-ICU, na: not apply.

| *Acinetobacter baumannii* | | | | |
| --- | --- | --- | --- | --- |
| Antibiotic | Localization | Susceptible | Non-susceptible | Fisher exact test |
| SAM | ICU | 12 | 41 | 0.2139 |
| SAM | INX | 43 | 88 |  |
| IPM | ICU | 7 | 26 | 0.8089 |
| IPM | INX | 19 | 58 |  |
| MEM | ICU | 11 | 41 | 0.2027 |
| MEM | INX | 40 | 87 |  |
| AK | ICU | 12 | 25 | 0.8326 |
| AK | INX | 26 | 61 |  |
| CIP | ICU | 11 | 41 | 0.4527 |
| CIP | INX | 35 | 92 |  |
| SXT | ICU | 6 | 31 | 0.165 |
| SXT | INX | 22 | 52 |  |

SAM: ampicillin-sulbactam, IPM: imipenem, MEM: meropenem, AK: amikacin, CIP: ciprofloxacin, SXT: trimetoprim-sulfamethoxazole. ICU: intensive care unit, INX: hospitalized non-ICU.

| *P. aeruginosa* | | | | |
| --- | --- | --- | --- | --- |
| Antibiotic | Localization | Susceptible | Non-susceptible | p (chi^2^) |
| CAZ | ICU | 59 | 46 | 0.005 |
| CAZ | OUT | 118 | 39 |  |
| CAZ | EME | 72 | 24 |  |
| CAZ | INX | 343 | 164 |  |
| TZP | ICU | 27 | 30 | <0.01 |
| TZP | OUT | 69 | 16 |  |
| TZP | EME | 43 | 16 |  |
| TZP | INX | 194 | 107 |  |
| FEP | ICU | 65 | 43 | 0.003 |
| FEP | OUT | 125 | 34 |  |
| FEP | EME | 79 | 20 |  |
| FEP | INX | 369 | 146 |  |
| CIP | ICU | 63 | 45 | 0.387 |
| CIP | OUT | 88 | 69 |  |
| CIP | EME | 61 | 38 |  |
| CIP | INX | 330 | 192 |  |
| IPM | ICU | 26 | 35 | 0.006 |
| IPM | OUT | 60 | 30 |  |
| IPM | EME | 41 | 19 |  |
| IPM | INX | 196 | 107 |  |
| MEM | ICU | 52 | 53 | <0.01 |
| MEM | OUT | 91 | 39 |  |
| MEM | EME | 70 | 25 |  |
| MEM | INX | 287 | 208 |  |

CAZ: ceftazidime, TZP: piperacillin-tazobactam, FEP: cefepime, CIP: ciprofloxacin, IPM: imipenem, MEM: meropenem, ICU: intensive care unit, OUT: outpatients, EME: emergency, INX: hospitalized non-ICU, na: not apply.

| *S. aureus* | | | | |
| --- | --- | --- | --- | --- |
| Antibiotic | Localization | Susceptible | Non-susceptible | p (chi^2^) |
| OXA | ICU | 48 | 17 | 0.262 |
| OXA | OUT | 95 | 19 |  |
| OXA | EME | 100 | 26 |  |
| OXA | INX | 318 | 104 |  |
| FOX | ICU | 38 | 13 | 0.438 |
| FOX | OUT | 80 | 19 |  |
| FOX | EME | 75 | 19 |  |
| FOX | INX | 228 | 80 |  |
| GN | ICU | 31 | 4 | 0.992 |
| GN | OUT | 52 | 8 |  |
| GN | EME | 34 | 5 |  |
| GN | INX | 150 | 23 |  |
| CIP | ICU | 51 | 9 | 0.174 |
| CIP | OUT | 86 | 21 |  |
| CIP | EME | 93 | 15 |  |
| CIP | INX | 300 | 87 |  |
| LVX | ICU | 47 | 11 | 0.686 |
| LVX | OUT | 97 | 17 |  |
| LVX | EME | 107 | 22 |  |
| LVX | INX | 341 | 83 |  |
| CC | ICU | 45 | 17 | 0.378 |
| CC | OUT | 82 | 25 |  |
| CC | EME | 99 | 22 |  |
| CC | INX | 309 | 105 |  |
| E | ICU | 51 | 13 | 0.204 |
| E | OUT | 79 | 31 |  |
| E | EME | 101 | 27 |  |
| E | INX | 297 | 121 |  |
| LZD | ICU | 59 | 1 | >0.05 |
| LZD | OUT | 110 | 4 |  |
| LZD | EME | 131 | 0 |  |
| LZD | INX | 434 | 1 |  |
| VN | ICU | 64 | 0 | na |
| VN | OUT | 114 | 0 |  |
| VN | EME | 123 | 0 |  |
| VN | INX | 391 | 0 |  |
| TE | ICU | 50 | 1 | 0.403 |
| TE | OUT | 96 | 6 |  |
| TE | EME | 92 | 2 |  |
| TE | INX | 299 | 17 |  |

OXA: oxacillin, FOX: cefoxitin, GN: gentamicin, CIP: ciprofloxacin, LVX: levofloxacin, CC: clindamycin, E: erythromycin, LZD: linezolid, VN: vancomycin, TE: tetracicline, ICU: intensive care unit, OUT: outpatients, EME: emergency, INX: hospitalized non-ICU, na: not apply.
